# A re-evaluation of the ‘quantile approximation method’ for random effects meta-analysis

**DOI:** 10.1002/sim.3487

**Published:** 2008-11-17

**Authors:** Dan Jackson, Jack Bowden

**Affiliations:** MRC Biostatistics Unit, Institute of Public HealthRobinson Way, Cambridge CB2 2SR, U.K.

**Keywords:** meta-analysis, random effects model, quantile approximation method

## Abstract

The quantile approximation method has recently been proposed as a simple method for deriving confidence intervals for the treatment effect in a random effects meta-analysis. Although easily implemented, the quantiles used to construct intervals are derived from a single simulation study. Here it is shown that altering the study parameters, and in particular introducing changes to the distribution of the within-study variances, can have a dramatic impact on the resulting quantiles. This is further illustrated analytically by examining the scenario where all trials are assumed to be the same size. A more cautious approach is therefore suggested, where the conventional standard normal quantile is used in the primary analysis, but where the use of alternative quantiles is also considered in a sensitivity analysis. Copyright © 2008 John Wiley & Sons, Ltd.

## 1. INTRODUCTION

Meta-analysis, the statistical process of combining the results from separate trials concerned with the same treatment or issue, is frequently used in medical statistics. A standard model for performing meta-analyses is the random effects model and the most commonly used procedure for implementing this is that suggested by DerSimonian and Laird [1]. Despite this it should be noted that other approaches are possible when using this model [2–4].

The random effects model assumes that the outcome from the *i*th of *k* trials is distributed as 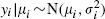, where μ_*i*_ is the true underlying treatment effect for this trial and 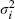 is the corresponding within-study variance. This variance is estimated in practice, but assumed fixed and known when pooling the results, and can be obtained for a range of measures used in meta-analysis as described in detail by Sutton *et al.* [5]. This conditional distribution is justified by the Central Limit and Slutsky's theorems but requires sufficiently large trials in order to provide a suitable approximation. The random effects model further assumes that μ_*i*_ ∼ N(μ, τ^2^), where μ and τ^2^ denote the overall treatment effect and between-study variance, respectively, and that the trials are independent. This provides the marginal distributions 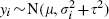.

DerSimonian and Laird estimate τ^2^ using the *Q* statistic,


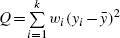


where 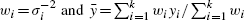. Evaluating *E[Q]* as a function of τ^2^ and matching this moment provides the DerSimonian and Laird estimate


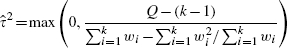


If 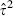 is truncated to zero then the resulting confidence intervals and results from hypothesis tests are the same as those from a fixed effects analysis, which assumes from the outset that 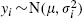. Hence 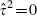 effectively means that a fixed effects procedure is adopted, although a random effects perspective may be maintained when interpreting the results.

Since 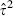 is a consistent estimate, *assuming that the number of trials is sufficiently large* we can, as a further approximation, replace τ^2^ by this estimate when making inferences about the overall treatment effect μ. Hence we use the model 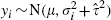 for this purpose, which results in


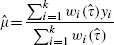
(3)

where 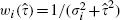. The variance of this estimate is approximately 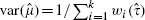 so that a nominal 95 per cent confidence interval is given by 
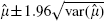
. However this relies on the notion that, approximately,


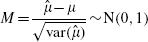


A recently proposed modification to the approach suggested by DerSimonian and Laird is the ‘quantile approximation method’ [6]. This ultimately follows a very similar procedure as DerSimonian and Laird but uses an alternative set of quantiles to the usual standard normal 0.975 quantile of 1.96 when constructing 95 per cent confidence intervals. These quantiles are derived from a simulation study using parameters that correspond to ‘typical scenarios for estimating a log odds ratio’ [6]. The resulting quantiles are all larger than 1.96 and the modification results in wider confidence intervals with greater coverage probabilities that are intended to correspond more closely to the nominal value than the more usual procedure achieves. A related argument that leads to the use of larger quantiles is the observation by Follmann and Proschan [7] that if the within-study variances are all equal, and one uses the untruncated version of the DerSimonian and Laird estimate, the resulting test statistic has a *t* rather than a standard normal null distribution. Sutton and Higgins [8, p. 628] refer to other recent studies that suggest using a *t*, rather than standard normal, null distribution in the context of meta-analysis. In particular, Sidik and Jonkman [9] suggest using a *t* distribution with a variety of variance estimators and emphasize that both the within and between-study variances are estimates. Bockenhoff and Hartung [10] address the specific issue of estimating the within-study variances, but here it will be assumed that the studies are large enough for these to be regarded as known and that the random effects model is correct. The problems are therefore caused by having to estimate the between-study variance, and using this as if it was the true value, in the conventional way.

Although the quantile approximation method is intuitively appealing, a single simulation study provides little assurance that the properties of *M* will be similar under alternative circumstances. The intention of this paper is to re-assess this method. The rest of the paper is set out as follows. In Section 2 the procedure from which the quantile approximation method was derived is briefly described, and in Section 3 some further simulation studies are performed, and analytical results are derived, that show that the original simulation study need not provide a good indication of the properties of *M* for alternative scenarios. A more cautious approach is therefore suggested, where the usual standard normal quantile is used in the primary analysis, but the use of alternative quantiles is considered in a secondary or sensitivity analysis. This procedure is illustrated using an example data set in Section 4. Section 5, the discussion, concludes the paper.

## 2. THE QUANTILE APPROXIMATION METHOD

The quantile approximation method has been recently proposed by Brockwell and Gordon; for a full description of this and its justification, see their original paper [6]. In the original simulation study, values of 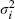 were simulated from a scaled and truncated χ^2^ distribution (with one degree of freedom, using a scale factor of 0.25 and then truncated to lie in the interval [0.009,0.6]). For each of *k* = 2,3, …,30, 19 values τ^2^ = 0,0.01,0.02,…, 0.1,0.15,0.2,…,0.5 were used, for each of which 25 000 simulated data sets were produced from the random effects model 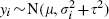 μ = 0.5 was used but this choice is immaterial. This results in 19 × 25000 = 475000 simulated meta-analyses for each *k*. The random variable 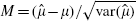 was evaluated for each of these sets of 475 000 meta-analyses and the 0.025 and 0.975 quantiles, denoted by Ĉ_0.025_ and Ĉ_0.975_, were obtained. Assuming symmetry, 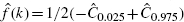 provides an estimate of the 0.975 quantile for any given *k*. In order to smooth the resulting quantiles, the 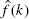 were regressed on 1/*k*, 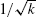 and 1/log(*k*) and this regression curve was used to provide the quantiles of *M*. Brockwell and Gordon propose that these quantiles be used when constructing 95 per cent confidence intervals, rather than the more conventional standard normal quantile of 1.96, and tabulate these values in their Table II. Other than this change in the quantile used, the quantile approximation method adopts the approach of DerSimonian and Laird in its entirety.

## 3. ADDITIONAL SIMULATION STUDIES AND ANALYTICAL RESULTS

In order to explore the applicability of the quantiles provided, two similar but slightly different simulation studies will be performed. These use exactly the same parameter values as those utilized by Brockwell and Gordon, except that the within-study variances will be truncated to lie in the intervals [0.06,0.6] and [0.0009,0.6], respectively. The first scenario is similar to the one explored previously, but with the important difference that large (small within-study variance) trials are not observed. Meta-analyses based on several relatively small trials are commonplace, and is the scenario, which this simulation study attempts to imitate. Since the 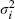 are simulated for every single trial in the simulation study, 475000*k* within-study variances are needed for each of *k* = 2, 3, …, 30, and a very large number of within-study variances is needed in total. The variances were simulated directly from a scaled 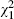 distribution and values that lay out of the allowable range of [0.06,0.6] were discarded. If the range were restricted further then it would be considerably more efficient to simulate directly from the distribution of the within-study variances.

The second scenario is also similar to that assumed by Brockwell and Gordon but permits very large ‘mega-trials’, which may occur in situations where such trials have been performed where these are deemed necessary in order to try to obtain a definitive estimate of treatment effect.

**Figure 1:**
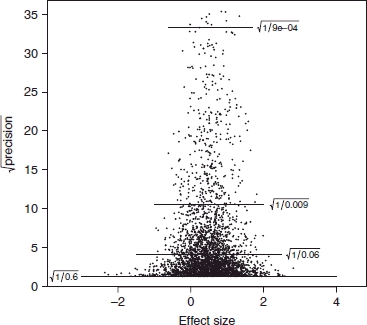
Funnel plot showing the trial sizes permitted by three alternative simulation studies.

The funnel plot shown in Figure 1 illustrates the types of trials permitted by these simulation studies. Here 3000 within-study variances have been simulated from the scaled χ^2^ distribution described above, but truncated to lie within [0.0008,0.6], from which indicative *y_i_* have been simulated from 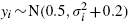. Here the precision is defined as 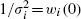 and the square-root of the precision is plotted against *y_i_*. The first simulation study performed here only permits small trials that lie beneath the horizontal line labelled 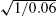, whereas the simulation study performed by Brockwell and Gordon permit those that lie beneath 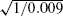. Finally the second simulation performed here permits trials that lie under 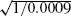 and allows very large mega trials as shown in Figure 1. The within-study variances used in Figure 1 were truncated to lie within [0.0008, 0.6] to emphasize that even the second simulation study described above does not permit trials of inordinate size, and the axes chosen were adopted to adequately display the variation in the trial sizes.

**Figure 2:**
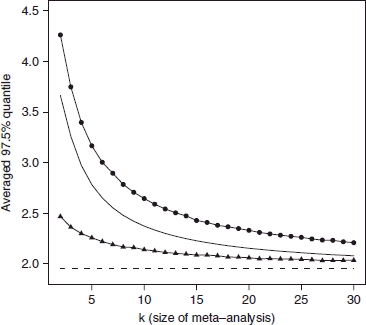
97.5 per cent quantiles of *M* from the simulation studies. Triangles show the results for the first scenario, where within-study variances lie in the interval [0.06,0.6]; circles show the results for the second scenario, where the within-study variances lie in the interval [0.0009,0.6]. The corresponding solid curves show the fitted regressions and the other solid curve follows the quantiles from the quantile approximation method. The dashed line shows the standard normal quantile, for comparison.

The results from the simulation studies are shown in Figure 2. For the first simulation study the plotted points, shown as triangles, are the simulated 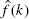 and the corresponding curve shows the fitted regression of 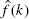 on *k*; the fitted model is 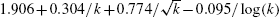.

The results from the second simulation study are similarly shown in Figure 2 using circles as plotting points. Here the fitted model is 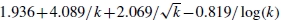 and much larger quantiles result. The other solid line follows the quantiles given by Brockwell and Gordon in their Table II, and the dashed line shows the standard normal quantile of 1.96 for comparison.

It is clear from Figure 2 that the distribution used for the within-study variances can have considerable implications for the resulting quantiles of *M*. The asymptotic results that justify the standard procedure ensure that, as *k* → ∞, the quantiles tend towards 1.96. Regression equations that have this limit might be considered, as the fitted regression equations do not provide this and should not be extrapolated for much larger values of *k*. We have not investigated this further, as this exercise merely serves to highlight that alternative quantiles result from different scenarios, and hence no such regression equation can give reliable quantiles for all circumstances.

### 3.1. Why do the simulation studies provide such different results?

The scenarios examined above were motivated by a consideration of Higgins and Thompson's [11] typical within-study variance, 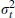. This is given by


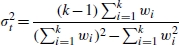


This formula applies to a finite sample of within-study variances, but in our simulation studies we assume a population distribution for these. Taking the limit as *k* → ∞ however provides this population and 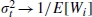 where *E[W_i_]* denotes the expectation of the reciprocal of the within-study variances. If the within-study variances are drawn from 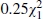, and then truncated to lie within [*a, b*], then the density of their reciprocal is


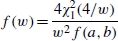


for *w* ∈ [1/*b*, 1/*a*] where 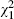 (·) denotes the probability density function of a χ^2^ distribution with one degree of freedom, and *f(a, b)* denotes the probability that this random variable lies in the interval [4*a*, 4*b*]. Hence


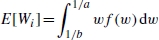


and the reciprocal of this integral gives the typical within-study variance for the population. For the simulation study conducted by Brockwell and Gordon, this typical within-study variance is 0.056.

The first simulation study however does not permit within-study variances less than 0.06, and the typical within-study variance is 0.160. For the second simulation study the typical variance is a mere 0.017. These very different typical study sizes help to explain the impact of the changes in the procedure and the difficulty in generalising findings from a particular simulation study to meta-analysis more generally, as the results depend on the value of τ^2^ in relation to the sizes of the studies, as demonstrated analytically in the next section.

### 3.2. The analytical form of M for the case where all trials are the same size

In order to further examine distribution of *M*, we will consider the special case where 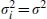 for all *i*, i.e. all trials are of the same ‘size’.

Under this simplification we have that the *y_i_* are i.i.d. from a normal distribution and therefore all the usual standard results apply. In particular the distribution of the sample mean of the *y_i_*, 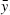, and the corresponding sample variance, 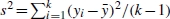, are related to those of well-known distributions and are independently distributed. Furthermore, the untruncated DerSimonian and Laird estimate of τ^2^ has the very simple form 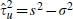. As the sample variance *s*^2^ is a continuous random variable, and hence the probability that it takes the value σ^2^ is precisely zero, there are therefore two distinct possibilities for the more usual estimate 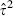 : either this is truncated to zero (if *s*^2^<σ^2^, so that a fixed effects model is adopted) or positive and equal to 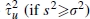. Let *E* denote an indicator random variable for the event that a random effects procedure is adopted, i.e. *E* = 0 if 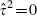 and *E* = 1 otherwise. We therefore have that the distribution of *M* is given by


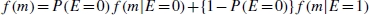
(1)

In order to obtain the distribution of *M* we evaluate the three expressions on the right hand of (1). These terms may be derived in a very similar manner as shown by Jackson [12], where the distribution of *M*, with μ = 0, is derived under the hypothesis *H*_0_: μ = 0. Defining the incomplete gamma function as


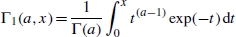


where Γ (·) denotes the usual Gamma function, we obtain


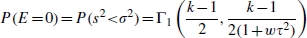
(2)

where *w* = σ^−2^. Conditioning on *E* = 0, and noting that 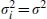 for all *i* gives 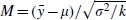. Noting that *E* = 0 is equivalent to the event that *s*^2^<σ^2^, and that 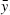 and *s*^2^ are independent, we obtain


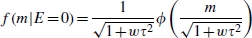
(3)

where φ(·) denotes the standard normal density function. We show in the appendix that


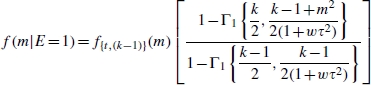
(4)

where *f*_(*t*ν)_(·) denotes the density of Student's *t* distribution with ν degrees of freedom. Substituting (2), (3) and (4) into (1) provides the required density *f(m)*. The density of *M* depends only on *k* and *w*τ^2^, and can also be expressed in terms of *k* and *I*^2^ = τ^2^/(σ^2^ + τ^2^) = *w*τ^2^/(1 + *w*τ^2^), the proportion of the variation provided by between-study variation. Higgins and Thompson [11] define a corresponding statistic, denoted here as 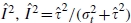. If 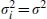 for all *i*, 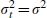 and *I*^2^ correspond to the true underlying value that Higgins and Thompson's statistic describes.

**Figure 3:**
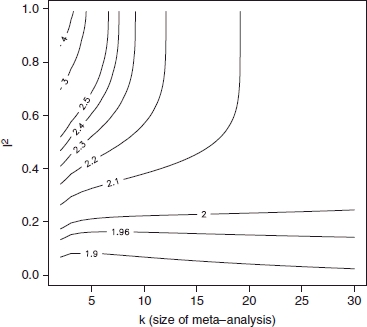
97.5 per cent quantiles of *M* assuming that all trials are of the same size.

As *f(m)* is an even function, for a fixed *k* and *I*^2^ the 0.975 quantile of *M* can be obtained numerically as the root of the equation


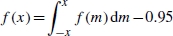


and a contour plot of these quantiles is shown in Figure 3. Note that the usual procedure of truncating 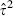, so that this is nonnegative, has been adopted. For the special case considered, if the untruncated version of 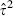 were used, *M* would follow a *t* distribution and the quantiles tend towards *t* quantiles as *I*^2^ → 1 in Figure 3. The quantile required is generally greater than the conventional 1.96 and for moderate *k* is highly sensitive to the value of *I*_2_. If the degree of heterogeneity is mild the appropriate quantile is slightly less than the usual standard normal quantile.

The special case considered in this section illustrates that the appropriate quantile depends on the degree of heterogeneity in relation to the size of the trials. It is therefore at the very least extremely difficult to ascertain an appropriate 0.975 quantile of *M* for meta-analysis in any degree of generality, which is precisely what the quantile approximation method professes to do.

### 3.3. A suggested procedure

With the above findings and discussion in mind, we make the following suggestion:

A primary analysis, using the conventional standard normal quantile of 1.96, is appropriate as this value is usual and justified asymptotically. For *k*<30 in particular however, the simulation studies and Figure 3 show that this may be too small in application and hence that nominal 95 per cent confidence intervals can provide somewhat smaller coverage probabilities. In particular, Î^2^ can be evaluated as 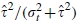 and Figure 3 can then be used as a rough guide to assess if the conventional quantile is likely to be appropriate. We further suggest computing a confidence interval for τ^2^ [2, 4, 13, 14], which can easily be transformed to provide a corresponding interval for *I*^2^. A range of plausible values of *I*^2^ may then be considered when inspecting Figure 3 and determining whether or not a secondary or sensitivity analysis is likely to be necessary.

The secondary analysis should involve a simulation study, using the observed within-study variances for the particular meta-analysis in question, and a range of τ^2^ that lie in the confidence interval, in order to investigate the appropriate quantile for *M*. We propose that the largest quantile for *M* uncovered by this simulation study should be used in the secondary analysis. For example, if a statistically significant result is overturned by this process, then extreme caution needs to be exercised when interpreting the primary analysis.

## 4. EXAMPLE: GLYCEROL FOR ACUTE STROKE

This example has previously been used by Jackson [15] and Baker and Jackson [16]. It concerns the use of glycerol for preventing death in patients who suffer an acute stoke, and involves nine trials with the results summarized as two by two tables. The data are shown in Table I, where *y_i_* denotes the log odds ratio of the *i*th trial and 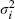 is the corresponding within-study variance, obtained in the usual way [5]; a negative log odds ratio indicates that the use of glycerol is beneficial.

**Table I:** The glycerol data.

Trial (*i*)	*y_i_*	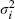
1	0.31	0.54
2	−0.57	0.17
3	0.01	0.62
4	0.38	0.24
5	0.21	0.39
6	−1.11	0.16
7	1.26	2.77
8	−0.20	0.09
9	0.36	0.23

The DerSimonian and Laird estimate of τ^2^ is 0.08. Using this estimate in a conventional random effects meta-analysis as described in Section 1 provides a 95 per cent confidence interval for μ of (−0.55,0.22). The analysis is therefore inconclusive, but if this is valid it does allow us to infer that the use of glycerol is neither particularly beneficial nor harmful. A 95 per cent confidence interval for τ^2^, using Viechtbauer's Q-profile method [14], is (0,0.95).

The typical within-study variance is 0.25 and a 95 per cent confidence interval for *I*^2^ is (0,0.79). Since *k* = 9, Figure 3 suggests that much larger quantiles than the standard 1.96 are plausible and might be considered in a secondary analysis. Six simulation studies were therefore performed using the empirical distribution of within-study variances, with τ^2^ = 0, 0.2, 0.4, 0.6 and 0.8 and 0.95. These values lie within the confidence interval for τ^2^ and cover a wide range of plausible possibilities. For each of these τ^2^, 25 000 meta-analyses involving nine trials with the 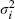 shown in Table I were simulated from the random effects model 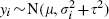; μ = 0.5 was again adopted although this choice is immaterial. Following the same procedure as before, *M* was computed for each simulated meta-analysis and the 0.025 and 0.975 quantiles were used to estimate the 0.975 quantile, resulting in quantiles of 1.853, 2.252, 2.389, 2.410, 2.389 and 2.385, for τ^2^ = 0, 0.2, 0.4, 0.6, 0.8 and 0.95, respectively. A worst case scenario is that a quantile of 2.410, rather than the standard 1.96, should be used when constructing the 95 per cent confidence interval for μ, which means that the usual interval should be widened by around 2.410/1.96≈23 per cent. This indicates that there is somewhat more uncertainty in the true treatment effect than the usual procedure suggests. For comparison, the quantile approximation method [6] gives the even larger quantile of 2.422, a value that does not seem particularly appropriate for the primary analysis for this particular example.

## 5. DISCUSSION

The usual random effects model for meta-analysis makes a series of assumptions and approximations. The first of these concerns the conditional normal distribution for *y_i_* | μ_*i*_ with known variance. This is a justifiable approximation provided that the trials are sufficiently large and this has been assumed throughout.

The normal model for the random effect is hard to verify empirically with typically small numbers of trials. In part due to the mathematical tractability of this assumption however, this is a fairly natural assumption to make. Assuming that the trials are independent is reasonable as they are conducted separately.

The final approximation, and one that is less likely to be appropriate in practice however, is the use of τ^2^ = 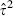 in the conventional way. This is only justified as the number of trials becomes large and the distribution of *M* may not closely follow a standard normal for the small sample sizes frequently encountered in practice. Despite this, under the random effects model, the density of *M* is not a function of μ: noting that *Q* is location invariant, 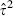 and therefore *M* can be written as a function of the *k* variables 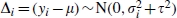, whose distributions do not depend on μ. Since the number and size of trials are clearly known, the only unknown variable that the distribution of *M* depends on is τ^2^. When assuming particular values of this in the context of a sensitivity analysis, *M* becomes a pivotal quantity for the remaining parameter μ. Hence *M* can be used to construct confidence intervals for the overall treatment effect using this pivotal quantity in the way proposed, for a range of values of τ^2^, after obtaining the appropriate quantiles approximately from simulation. The dependency of the quantile on τ^2^ is mentioned by Brockwell and Gordon, who discuss a quantile approximation function *g*(*k*, τ^2^). Our simulation studies highlight that this is also a function of the 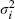, and Figure 3 shows that *g*(*k*, τ^2^) is sensitive to the value of τ^2^. The uncertainty in this value should be taken into account when deciding which quantiles to use.

Figure 3 illustrates the scenarios where the standard normal quantile provides a poor approximation, but is no substitute for obtaining quantiles by simulation, because this figure only takes into account the size of studies and considers a simple special case. An improvement might be to produce a series of figures akin to Figure 3 where both an average and a measure of variability of the study sizes is reflected in the quantiles plotted. These might be obtained from further calculations, if a suitable distribution of study sizes could be found, or more likely from additional simulation studies. Deriving the exact distribution of *M*, in full generality, remains one of the most important unsolved problems in meta-analysis.

Although the quantile approximation method attempts to take into account the exact distribution of *M* for finite sample sizes, its ‘one size fits all’ approach can be misleading. We propose that the type of sensitivity analysis illustrated in Section 4 using the glycerol data be used in its stead. This requires more work and attention to detail, but is relatively straightforward to implement nonetheless.

## APPENDIX A: PROOF OF EQUATION (4)

Conditioning on *E* = 1, and noting that  for all *i*, provides  and . A little rearrangement gives

where *X*_1_ and *X*_2_ are independent. Furthermore , conditional on *E* = 1, which is equivalent to the event that *X*_2_≥(*k* − 1)/(1 + *w*τ^2^)=*c*. The density *f*(*x*_1_, *x*_2_|*E* = 1) is given by *f* (*x*_1_) *f*(*x*_2_) *I*_(*x*_2_≥*c*)_/ *P* (*X*_2_≥*c*), where *I*_(*x*_2_≥*c*)_ = 1 if *x*_2_≥*c*) and *I*_(*x*_2_≥*c*)_ = 0 otherwise. Furthermore *P* (*X*_2_≥*c*) is given by the complement of equation (2). Changing variables in the usual way with  and *M*_2_ = *X*_2_, and integrating out *M*_2_ provides (4).
